# Inhibition of Chikungunya Virus Replication by 1-[(2-Methylbenzimidazol-1-yl) Methyl]-2-Oxo-Indolin-3-ylidene] Amino] Thiourea(MBZM-N-IBT)

**DOI:** 10.1038/srep20122

**Published:** 2016-02-04

**Authors:** Priyadarsee Mishra, Abhishek Kumar, Prabhudutta Mamidi, Sameer Kumar, Itishree Basantray, Tanuja Saswat, Indrani Das, Tapas Kumar Nayak, Subhasis Chattopadhyay, Bharat Bhusan Subudhi, Soma Chattopadhyay

**Affiliations:** 1 School of Pharmaceutical Sciences, Siksha O Anusandhan University, Bhubaneswar, India; 2 Institute of Life Sciences, Bhubaneswar, India; 3 School of Biological Sciences, National Institute of Science Education & Research, Bhubaneswar, India

## Abstract

Chikungunya virus (CHIKV) infection is one of the most challenging human Arboviral infections with global significance and without any specific antiviral. In this investigation, 1-[(2-methylbenzimidazol-1-yl) methyl]-2-oxo-indolin-3-ylidene] amino] thiourea (MBZM-N-IBT) was synthesised as a molecular hybrid of 2-methyl benzimidazole and isatin-β-thiosemicarbazone and its anti-CHIKV property was evaluated. The release of infectious virus particles was calculated by plaque assay, expression profile of viral RNA was estimated by RT-PCR and viral protein profiles were assessed by Western blot and FACS analyses. The safety index of MBZM-N-IBT was found to be >21. The CHIKV infectious viral particle formation was abrogated around 76.02% by MBZM-N-IBT during infection in mammalian system and the viral RNA synthesis was reduced by 65.53% and 23.71% for nsP2 and E1 respectively. Surprisingly, the viral protein levels were reduced by 97% for both nsP2 and E2. In the time-of-addition experiment it abrogated viral infection at early as well as late phase of viral life cycle, which indicates about multiple mechanisms for its anti-CHIKV action. *In silico* analysis justified development of MBZM-N-IBT with good affinities for potential target proteins of CHIKV and related virus. With predictions of good drug-likeness property, it shows potential of a drug candidate which needs further experimental validation.

The name Chikungunya fever (CHIKF) derives from the Makonde language from Tanzania, Africa which means the “bend up”, characterizing the posture of the patients suffering from severe joint pain due to Chikungunya virus (CHIKV). First, it was isolated in 1952 from Tanzania^1^. CHIKV infection leads to silent incubation period of about 2 to 4 days which may range from 1 to 12 days also^2^. This disease is spread by *Aedes aegypti* and *Aedes albopictus* mosquitoes and symptoms are mainly high fever, polyarthralgia, myalgia, nausea, rashes^2^, photophobia^3^ and headaches^1^,^2^. In few cases neuronal complications have also been reported^4^, but the major clinical symptom is polyarthralgia which may persist for several months in few cases^5^. The 2005-06 outbreaks of CHIKV in the islands of the Indian Ocean^6^ and several other cases which were detected recently in the America have changed the notion that CHIKV is confined to Asian and African countries only.

CHIKV is one of the 30 species of Alphavirus genus, and belongs to the *Togaviridae* family^7^. This virus is spherical (approximately 70 nm diameter), enveloped with a 12 kb long positive sense single stranded RNA genome. The genome codes for two open reading frames, the first one (49S RNA) encodes for four non-structural proteins (nsP1-4) and the second one (26S RNA) encodes for three major structural proteins (C, E1 and E2)^8^.

Although, many groups have started working to develop effective vaccine or antiviral drugs for CHIKV infection, still there is no licenced vaccine or drug available. One promising attenuated live vaccine (TSI-GSD-218) was obtained after serial passaging, and was tested in human phase II trial by US army^9^. Studies regarding the development of CHIKV vaccine have also been reported where the recombinant vaccine or DNA encoding CHIKV structural proteins are utilized^10^.

Till now, Chloroquine, an antimalarial drug is used to manage chronic Chikungunya arthritis^11^. However, it is only effective in early stages of viral life cycle, which limits its use to prophylactic management only^12^. Ribavirin was demonstrated to inhibit RNA virus *in vitro*^13^. At a dose of 200 mg twice a day for 7 days, it showed a reduction in symptoms of CHIKV infection^14^ and with pegylated interferon it showed synergistic *in vitro* inhibition^15^. However, at this dose level it is known to be genotoxic and cytotoxic^16^ due to which its therapeutic efficacy is yet to be established against CHIKV infection. Among broad spectrum antiviral compounds, only arbidol has so far shown to possess some *in vitro* potential against CHIKV in primary human fibroblast and Vero cells^17^. Recently, efforts have been made to develop more potent analogues of arbidol against CHIKV^18^,^19^. However, their mode of action is not clear and further investigations are necessary for their optimization before clinical trials against CHIKV. Harringtonine, a cephalotaxine alkaloid was recently shown with *in vitro* antiviral activity against CHIKV^20^. Recently, silymarin and suramin were reported to have anti-CHIKV property *in vitro*^21^,^22^. However, these were active mostly on early phase of CHIKV life cycle which may limit their therapeutic utility post infection. Further evaluations are required *in vivo* for the establishment of their therapeutic application. Hence, there is no specific anti-CHIKV drug available due to which its management is still limited to symptomatic treatment with existing NSAIDS.

Isatin-β-thiosemicarbazone (IBT) was first demonstrated to have antiviral action against vaccinia virus in 1953 by Hamre *et al.*^23^. This encouraged further optimization^24^, that led to development of MIBT as a potent compound against vaccinia virus which was later shown to be clinically effective^25^. However, it had only limited efficacy in treatment of small pox and patients suffered from complications^26^. Besides, MIBT and its derivatives were found to be ineffective against many other virus including poliomyelitis, influenza (NWS strain), rabies (Flury strain), Ilheus, Wyeomyia, Zika, California, Choriomeningitis, Ntaya, Semliki, herpes, dengue 1, Anopheles A, Anopheles B^27^. These factors led to lack of further interest in MIBT. Nevertheless, MIBT has been used as a lead to develop more potent antiviral^28^,^29^. However, these efforts have not yet yielded any clinically effective antiviral. Considering this, as there is no report of MIBT efficacy against Alphavirus, we were interested to develop its derivative for evaluation against CHIKV.

Benzimidazoles are endowed with diverse pharmacological activities. Being isosteric with purine and indole nuclei, benzimidazole has been part of several bioactive compounds^30^. Derivatives of 2-methyl benzimidazole have been reported with antiviral activity against virus including HCV, HBV, HIV-1, human RSV^31^,^32^, but 2-methyl benzimidazole itself has not been reported with any potent activity^33^. There is no report of its action against CHIKV. Considering the presence of 2-methyl benzimidazole moiety in these reported compounds, it was thought worthwhile to develop MBZM-N-IBT as a molecular hybrid with IBT for evaluation against CHIKV.

## Results

### Synthesis of compounds

**Figure 1:**
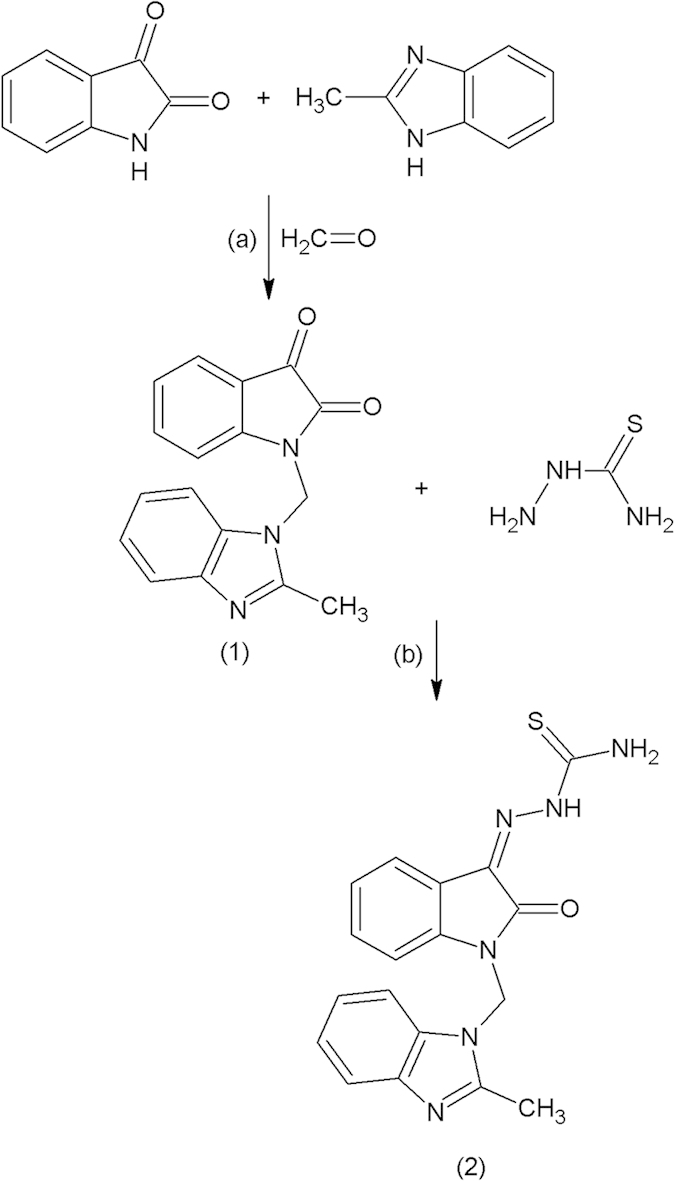
Synthesis of MBZM-N-IBT. **(a)** Isatin, paraformaldehyde, and 2-methyl benzimidazole were dissolved in ethanol and refluxed for 16h to yield the intermediate compound (1); **(b)** Compound (1) and thiosemicarbazide were dissolved in warm ethanol and refluxed for 4h following addition of few drops of glacial acetic acid to yield MBZM-N-IBT (2).

**Figure 2:**
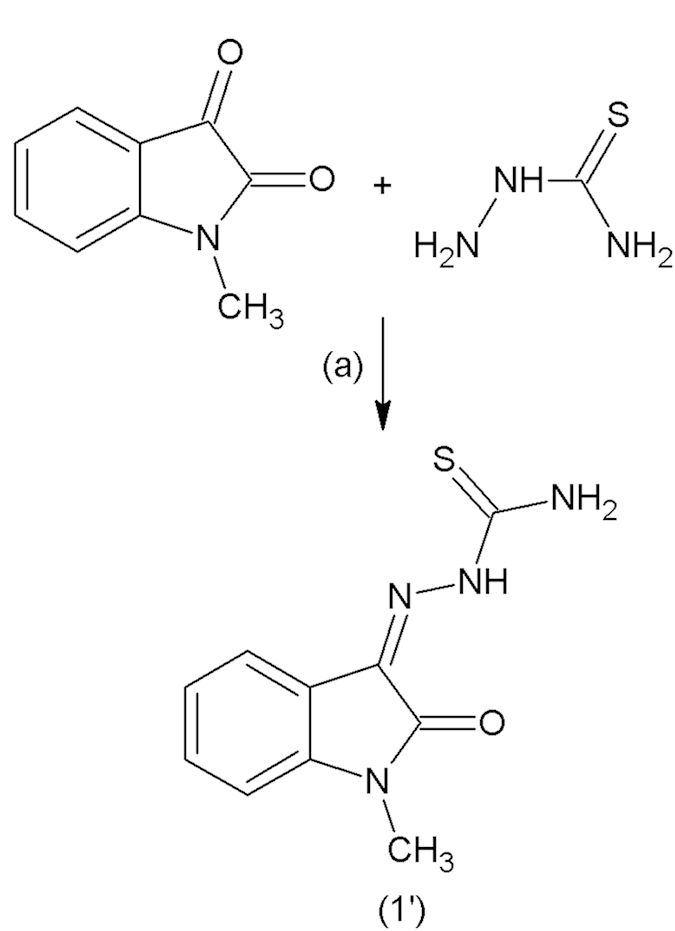
Synthesis of MIBT. N-methyl isatin and thiosemicarbazide were dissolved in warm ethanol and refluxed for 4h following addition of few drops of glacial acetic acid to yield MIBT (1′).

**Figure 3:**
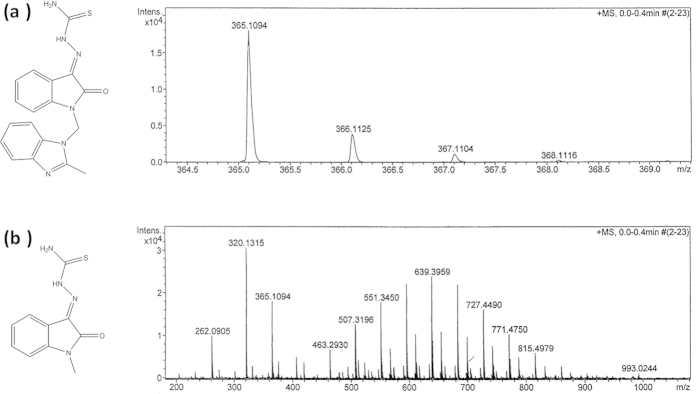
Structure and mass spectrum of (a) MBZM-N-IBT and (b) MIBT. Mass spectrum was recorded with ESI technique in micrOTOF-QII mass spectrometer.

The test compound MBZM-N-IBT and MIBT were synthesised as per the scheme in Fig. 1 and 2 with good yield. Purity of the compounds was checked in HPLC and it was found to be more than 95% (Supplementary Fig. S1a and S2a). MBZM-N-IBT was recovered as brownish solid with a melting point of 162 °C. The structure of compound (2) was proposed based on the spectral data as mentioned below (Supplementary Fig. S1b, S1c, and S1d).The mass spectrum of MBZM-N-IBT with M + 1 mass peak at 365 m/z (Fig. 3a) further confirmed this. Similarly, MIBT was recovered as brown colored compound with a melting point of 245 °C which matches with that of the reference (U.S National Library of Medicine, TOXNET). The compound (1′) showed characteristic spectral data (Supplementary Fig. S2b, S2c, and S2d) supported by M + 1 mass peak at 235 m/z (Fig. 3b), that confirmed formation of MIBT.

### Inhibition of CHIKV replication by MBZM-N-IBT

**Figure 4:**
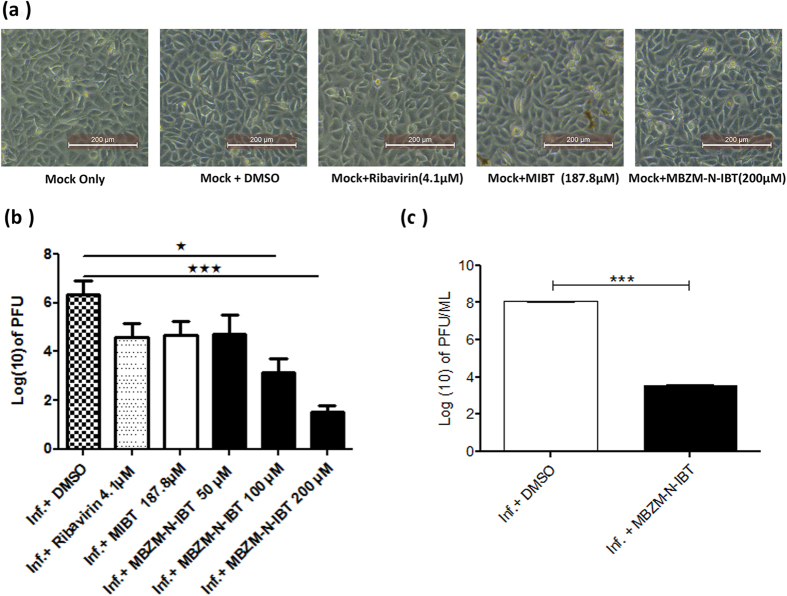
Inhibition of CHIKV infection by MBZM-N-IBT. Vero cells were infected with Chikungunya virus prototype strain S 27 (MOI 0.001). DMSO and different doses of the drugs [MBZM-N-IBT (50 μM), 100 μM and 200 μM)] were added to the experimental samples. DMSO was used as negative control and recent reported drugs Ribavirin (4.1 μM) and MIBT (187.8 μM) were used as positive control. The cells and supernatants were harvested at different time post infection based on the assay. **(a)** Morphological changes were observed under microscope at 15 hpi and pictures were taken with 20X magnification. **(b)** The supernatants as well as cells were collected from all the experimental samples at 15 hpi and plaque assay was performed to assess the number of infectious particle of CHIKV. **(c)** MBZM-N-IBT was added in DMEM complete media and kept in CO_2_ incubator at 37 °C for 15 hrs. The whole contents was added into the CHIKV infected Vero cells and virus titer was determined by plaque assay as described earlier. The bar diagram represents the virus titer in log (10) scale for all the experimental samples from three independent experiments. The statistical analysis of the experimental data was presented as mean ± SD of three independent experiments (n ≥ 3).P - value less than 0.001 was considered to be statistically significant in the tests (*p < 0.05; **p ≤ 0.01, ***p ≤ 0.0001).

To assess the effect of the drugs on Vero cells, the cells were treated with 50, 100 and 200 μM concentration of MBZM-N-IBT, MIBT (187.8 μM)^18^ and Ribavirin (4.1 μM)^20^ for 15 hrs and were observed under microscope for morphological changes. The Vero cells were treated with MIBT and Ribavirin as control because MIBT is the prototype of IBT class of compounds^34^ and Ribavirin was shown to have anti-CHIKV property previously^20^. The Fig. 4a showed that, there was no morphological change after the drug treatments which indicate that these drugs might not have cytotoxic effect with the tested dose level.

Next, the cytotoxicity of MBZM-N-IBT (50 to 800 μM) on Vero cells were estimated by MTT assay according to the method described here. It was observed that more than 82% cells were viable with 800 μM of MBZM-N-IBT, hence the CC50 value was considered to be >800 μM. Whereas, MIBT showed 82% cell viability with 50 μM and 35% cell viability with 800 μM (data not shown). As the drug was not cytotoxic to the cells with the concentration of 800 μM and time of exposure which we have followed, therefore dose-dependent inhibition of MBZM-N-IBT on CHIKV infection was carried out.

In order to estimate the reduction in mature infectious viral particle formation after treatment, the Vero cells were infected with CHIKV prototype strain S 27 with multiplicity of infection (MOI) 0.001. The CHIKV infected Vero cells were treated with 50, 100 and 200 μM concentration of MBZM-N-IBT to perform plaque assay. Cells were observed for cytopathic effect (CPE) under microscope. The Vero cells and supernatants were harvested at 15 hours post infection (hpi) after S 27 infection and treatment of MBZM-N-IBT (50, 100 and 200 μM), Ribavirin (4.1 μM) and MIBT (187.8 μM). The harvested samples were used to infect Vero cells and plaque numbers were counted after 3 to 4 days according to the procedure mentioned below. The plaque numbers were converted into log 10 of PFU/ml and plotted in the bar diagram (Fig. 4b). As shown in the Fig. 4b, it was observed that Ribavirin (4.1 μM) and MIBT (187.8 μM) reduced the plaques by 26.66% and 28.07% respectively in comparison to DMSO control. Whereas, the plaque numbers were decreased by 25.87%, 50%, and 76.02% after treatment with 50, 100, and 200 μM of MBZM-N-IBT respectively (Fig. 4b). To support the above observation, the viral RNA was extracted from the collected cells and supernatant and RT-qPCR was performed as mentioned in materials and method section. It was observed that the viral RNA was also decreased significantly (*p* < *0.05*) after MBZM-N-IBT treatment (Supplementary Fig. S3). However, the reduction in viral RNA was moderate as compared to reduction in infectivity. This is because all viral RNAs are not expected to result in infectivity which explains the remarkable reduction in plaque assay but moderate reduction in RT-qPCR.

Next, the EC_50_ value of MBZM-N-IBT was calculated for S 27 and it was found that EC_50_ is 38.68 μM (Supplementary Fig. S4a)

Next, we were interested to evaluate the antiviral effect of MBZM-N-IBT for another strain of CHIKV. Earlier, we have studied a 2006 Indian isolate, DRDE-06 and observed that this strain can replicate faster in comparison to S 27^35^. Hence, MBZM-N-IBT was added to the Vero cells after DRDE-06 infection and viral titer was determined. As mentioned above, the EC_50_ value for DRDE-06 was also determined and it was found to be 58.93 μM (Supplementary Fig. S4b) which is higher than that of S 27 strain. Earlier this was reported that because of higher and faster replication ability of DRDE-06, the inhibitory effect of Geldanamycin was less for this virus as compared to S 27^36^. Hence, the EC_50_ value difference between these two viruses is expected. It was noticed that the viral titer was reduced by 99.96% in presence of 200 μM MBZM-N-IBT (Supplementary Fig. S5) indicating that this is active against other CHIKV strain also.

Taken together, our result indicates that MBZM-N-IBT can inhibit CHIKV infection significantly (*p* < *0.001*). As it is not cytotoxic to the host cells, the inhibitory effect may be due to the specific antiviral activity of MBZM-N-IBT.

In order to assess the stability of MBZM-N-IBT in the 37^o^C incubator, MBZM-N-IBT was added in DMEM complete media and kept in CO_2_ incubator at 37 °C for 15 hrs. Next, the whole content was added into the CHIKV infected Vero cells and supernatant was collected after 15hpi. The virus titer was determined by plaque assay as described earlier. As shown in Fig. 4c, the pre-incubated MBZM-N-IBT was able to inhibit viral infection by 99.99%. which is found to be significant (*p < 0.05; **p  ≤ 0.01, ***p  ≤ 0.0001).

### MBZM-N-IBT reduces viral RNA and viral protein levels

**Figure 5:**
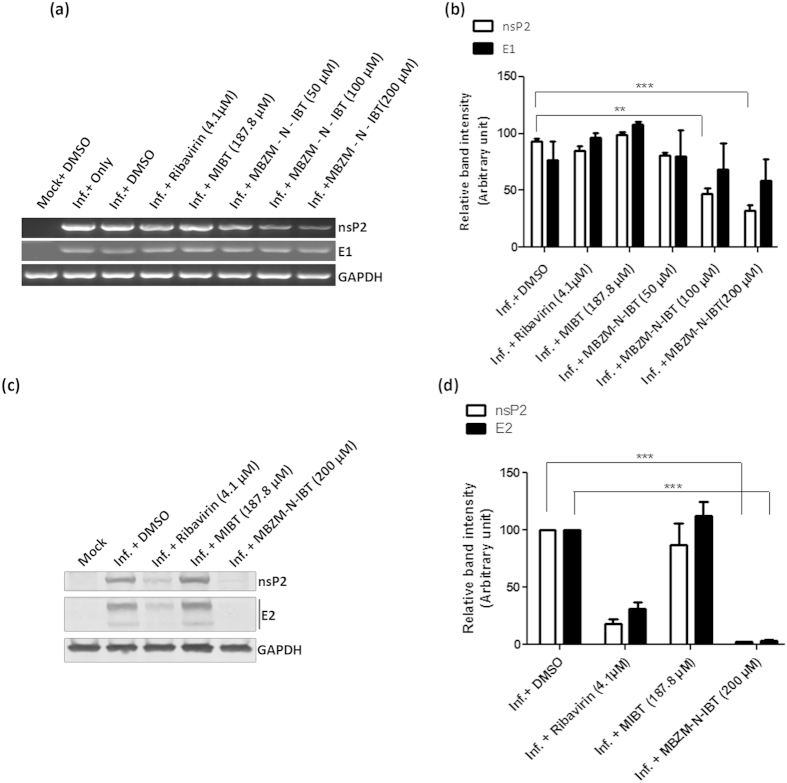
Effect of MBZM-N-IBT in CHIKV RNA and protein level. **(a)** Chikungunya virus (S 27) infected with MOI 0.001 and treated with DMSO (negative control), 4.1 μM Ribavirin (positive control), 187.8 μM MIBT and (50, 100, 200 μM) MBZM-N-IBT. Whole cell RNA was isolated from the CHIKV infected samples at 15hpi and CHIKV nsP2 and E1 genes were amplified using respective primers by RT PCR. **(b)** Bar diagrams showing the relative band intensities in viral RNA expression pattern in infected and drug treated samples as obtained through PRISM Software. Data represented as mean ± SEM from three independent experiments. p ≤ 0.05 was considered statistically significant. **(c)** As mention above infected Vero cells lysates were separated in 10% SDS PAGE and viral protein expression pattern were assessed by Western blot using antibodies against CHIKV nsP2 and E2 proteins. In both, GAPDH was used as a loading control. **(d)** Bar diagrams showing the relative band intensities in viral protein expression pattern in infected and drug treated samples as obtained through PRISM Software. Data represented as mean ± SEM from three independent experiments. p ≤ 0.05 was considered statistically significant.

It was noticed during our study that MBZM-N-IBT was capable of reducing the viral particle formation in mammalian cells. MBZM-N-IBT was added to the cells after viral infection process; hence, we were interested to understand the stage of CHIKV life cycle which might have been affected by this drug. To find out that, Vero cells were infected, drug treated and harvested at 15 hpi. Viral RNA levels were observed by RT-PCR and viral protein levels were assessed by Western blot and flow cytometry. As a representative of non-structural and structural proteins, nsP2 and E1/E2 were selected for the above mentioned methods. As shown in Fig. 5a, with the treatment of 100 and 200 μM of MBZM-N-IBT, the RNA level of nsP2 was reduced by 50.02% and 65.53%. Similarly, the RNA level for E1 was reduced by 10% and 23.71% respectively. Whereas, MIBT treatment to the virus infected Vero cells did not show reduction in the viral RNA levels. The level of RNA was quantified by normalizing the GAPDH amount and the relative band intensities of Fig. 5a has been plotted in Fig. 5b for comparison. Thus, our result indicates that the viral RNA level of non-structural protein was decreased significantly (*p* < *0.001*) in presence of MBZM-N-IBT.

In order to elucidate the effect of the drug on viral protein levels, Western blot and FACS analyses were carried out for the nsP2 as well as E2 proteins. It was observed that the nsP2 protein level was decreased by 97.53% in presence of 200 μM MBZM-N-IBT and E2 protein level was decreased by 97.5% (Fig. 5c,d). Interestingly, it was noticed that nsP1 and nsP3 viral protein levels were also reduced similarly after treatment (data not shown). On the other hand, MIBT treatment to the infected cells did not show any effect on viral protein levels. Surprisingly, the viral protein levels were reduced after Ribavirin treatment and a remarkable reduction was observed following MBZM-N-IBT treatment (*p* < *0.001*)(Fig. 5c,d).

**Figure 6:**
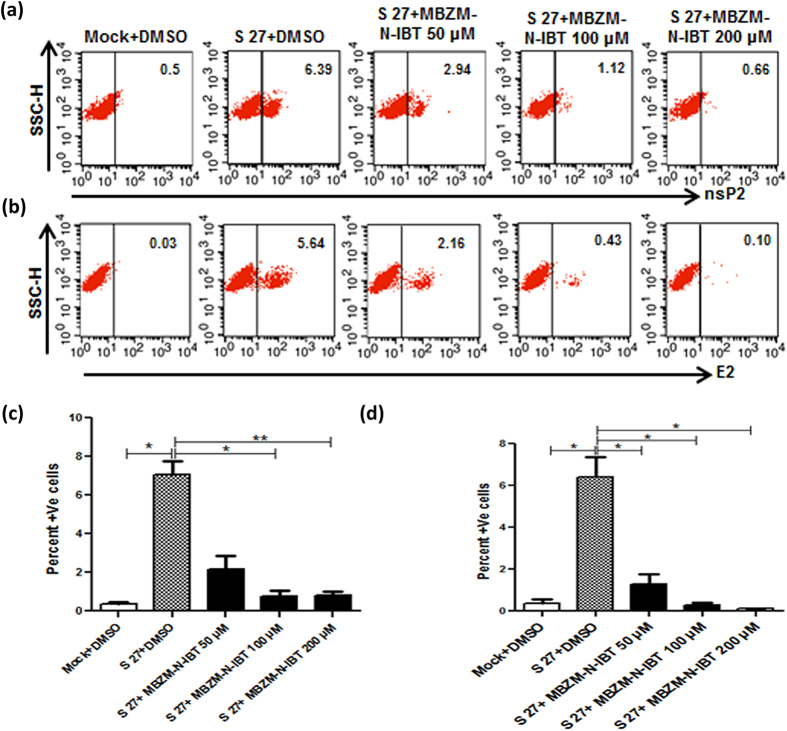
Flow cytometric analysis depicting inhibition of CHIKV nsP2 and E2 protein expression after treatment with MBZM-N-IBT. Dot plot analysis showing percent positive cells for nsP2 **(a)** and E2 **(b)** against SSC-H towards mock + DMSO, S 27 + DMSO, S 27 + MBZM-N-IBT (50, 100 and 200 μM). Graphical representation showing percent positive cells for nsP2 **(c)** and E2 **(d)** of S 27 + DMSO, S 27 + MBZM-N-IBT (50, 100 and 200 μM). Student’s t test was performed to calculate p values. p < 0.05 was considered statistically significant between groups (*p ≤ 0.05; **p ≤ 0.01).

Similar effect of the test compound on the viral proteins was also observed by Flow cytometric analysis. Virus infected and drug treated cells were processed for Flow cytometry as mentioned below. The number of cells showing nsP2 and E2 expression was reduced by 88.41% and 98.13% respectively (Fig. 6a–d). Moreover, the protein levels of nsP2 were reduced by 51.91% (Supplementary Fig. S6) and E2 level was reduced by 79.37% (Supplementary Fig. S6) after the treatment of 200 μM MBZM-N-IBT. Taken together, the results suggest that viral RNA levels are affected after MBZM-N-IBT treatment; however, the viral protein levels are reduced remarkably.

### Inhibition in infectious viral particle release (99%) even after addition of MBZM-N-IBT at 8 hpi

**Figure 7:**
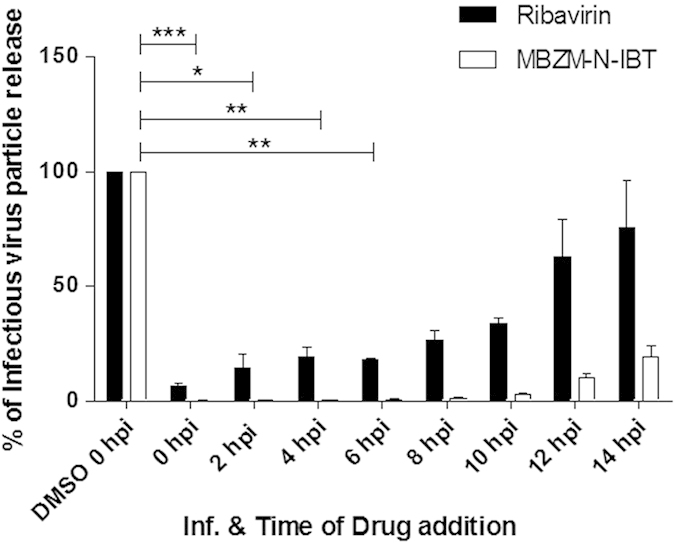
Inhibition pattern of CHIKV infection by addition of MBZM-N-IBT at different time points. Vero cells were infected by Chikungunya virus prototype strain S 27 with MOI 0.001 and 200 μM MBZM-N-IBT was added to each sample at every 2 hrs interval up to 14 hpi. Ribavirin (4.1 μM) was used as a control. The supernatants as well as cells were collected from all the experimental samples at 15 hpi and plaque assay was performed to assess the number of infectious particles of CHIKV. The bar diagram represents the virus titre in PFU/ML scale for all the experimental samples from three independent experiments. Open bar and close bar indicate the DMSO only and with MBZM-N-IBT respectively. The statistical analysis of the experimental data was presented as mean ± standard deviation of three independent experiments. P – value less than 0.001 was considered to be statistically significant in the test.

To find out the possible mechanism of action for the MBZM-N-IBT drug on CHIKV replication, a time of addition experiment was performed. The Vero cells were infected with CHIKV prototype strain S 27 with MOI 0.001 and 200 μM MBZM-N-IBT was administered at 0, 2, 4, 6, 8, 10, 12, and 14 hpi. Ribavirin (4.1 μM) was used as a control. Next, the CHIKV infected and MBZM-N-IBT post treated Vero cells and supernatants were harvested at 15 hpi and plaque assay was performed as mentioned above to assess the release of infectious virus particles. As shown in Fig. 7, it was observed that around 80% of the infectious virus particle release was abrogated in presence of 200 μM MBZM-N-IBT even after addition of the drug at 14hpi which indicate that a short exposure is sufficient to reduce virus release significantly. Interestingly, the release of infectious virus particles was inhibited by 99% with addition of MBZM-N-IBT at 0, 2, 4, 6, and 8 hpi as compared to DMSO treatment. Unlike MBZM-N-IBT, Ribavirin showed only 74% inhibition in viral particle release at 8 hpi which was reduced further at higher hpi and was less than 25% when Ribavirin was added at 14 hpi. This finding suggests that, MBZM-N-IBT might interfere in multiple stages of CHIKV life cycle.

### MBZM-N-IBT shows strong binding affinities for CHIKV proteins

Considering the possibility of multiple targets involved in MBZM-N-IBT action against CHIKV, a particular target cannot be assigned at this stage; however, to justify the selection of molecular hybrid of IBT and MBZM as MBZM-N-IBT for efficacy against CHIKV, molecular docking was adopted. Although, viral proteins cannot be considered as the only vital target, for convenience and taking clues from remarkable reduction of viral protein, both structural and non-structural proteins were selected for molecular docking study. The AutoDock Vina, an open-source program, was used for molecular docking^37^. Using this, molecular docking of MBZM-N-IBT and MIBT were carried out with structural and non-structural proteins of CHIKV. The binding affinity for MBZM-N-IBT against 3N41 and 3TRK was-7.4 Kcal/mol. whereas; MIBT showed binding affinities of −5.9 and −6.4 Kcal/mol against the same targets (Table 1). The decoys used in this investigation are different structural components of MBZM-N-IBT which includes isatin, IBT, 2-methyl benzimidazole and thiosemicarbazone. The binding affinities of decoys against 3N41 and 3TRK were less than MBZM-N-IBT (Table 1) which supports the fact that molecular hybridisation has enhanced the affinity for CHIKV targets.

**Figure 8:**
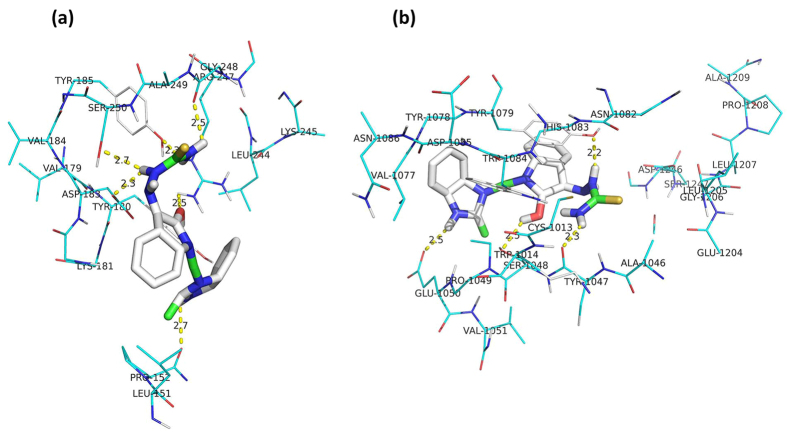
MBZM-N-IBT shows strong binding affinities for CHIKV proteins. The best fit complex of MBZM-N-IBT with, (**a**) mature envelope glycoprotein complex (spontaneous cleavage) of CHIKV (3N41) and (**b**) nsP2 protease of CHIKV (3TRK). MBZM-N-IBT was screened against the target protein in the AutoDock Vina open-source program for molecular docking and the best fit complex was visualised in the PyMOL viewer.

Further, the analysis of the most preferred binding mode of MBZM-N-IBT with structural protein (3N41) exhibited four polar interactions with thiosemicarbazone moiety at ASP183, SER 250, TYR 185, and GLY 248 residues (Fig. 8a). In addition, the carbonyl group of isatin moiety showed one polar interaction at ARG247 position and the benzimidazole moiety showed another polar interaction at the position of PRO152. Similarly, the analysis of the most preferred binding mode of MBZM-N-IBT with protease domain of nsP2 (3TRK) exhibited polar interactions of thiosemicarbazone with TYR 1047 and TYR 1079 (Fig. 8b). The benzimidazole moiety exhibited one polar interaction at GLU 1050 position. Another polar interaction was observed between isatin group and TRP1014. These groups were also closely surrounded by aromatic residues of TYR 1047, TYR 1079, TYR 1078, TRP 1014, TRP 1084 and HIS 1083, which may contribute to the π stacking and lead to enhanced stability of the complex. Besides, MBZM-N-IBT was found to be embedded in the catalytic dyad of nsP2 protease (HIS 1083, and CYS 1013).

Molecular docking of MBZM-N-IBT was also carried out against nsP1, nsP3, and nsP4 of CHIKV using earlier developed homologous models and it was noticed that MBZM-N-IBT exhibited binding affinities of −7.3, −7.9, and −8.3 Kcal/mol respectively which are higher than any decoys (Supplementary Table S1). This justifies the development of MBZM-N-IBT as potent antiviral molecule against CHIKV.

Next, the question was whether the potential of MBZM-N-IBT is specific to CHIKV. In order to address this, the binding affinities against other proteins of Alphaviruses (Semliki Forest Virus, 1RER; Sindbis virus, 3MUU) were estimated by *in silico* analysis. Molecular docking experiments revealed −8.5 and −7.6 Kcal/mol as binding affinities of MBZM-N-IBT against structural proteins of Sindbis and Semliki forest viruses.

## Discussion

Widespread incidences of CHIKV infection have encouraged researchers for the development of suitable vaccine and antiviral molecules for its management.

In this study, MBZM-N-IBT was synthesized by adapting established protocol and its anti-CHIKV efficacy was assessed. The purity of the compounds was more than 95% after synthesis. The MASS, NMR and FTIR data of the compound were in agreement with the proposed structure. It was observed that MBZM-N-IBT inhibited CHIKV infection significantly in Vero cells with minimum cytotoxicity. Our result revealed that CHIKV RNA levels (65.53% for nsP2 and 23.71% for E1) were reduced in the presence MBZM-N-IBT, however, CHIKV structural as well as non-structural protein levels were decreased almost by 97%. A time-of-addition experiment demonstrated that MBZM-N-IBT was capable of abrogating viral infection at early as well as late time of administration and a short exposure (one hour) of the compound was capable of reducing the viral titer significantly which is desirable for minimizing adverse effect of the drug candidate. Further, *in silico* analysis justified the development of MBZM-N-IBT as a molecular hybrid of MBZM and IBT, however the binding affinities require further experimental validation.

Since the first report of antiviral properties of thiosemicarbazones by Hamre *et al.*^23^, several attempts have been made to develop structurally related antivirals with little success. MIBT is the prototype of this class of compounds which was shown to have some clinical efficacy against Pox virus^34^. However, after an intake of 3 gm/day, it was only useful prophylactically and had no therapeutic effect against Variola infections and is known to cause nausea and vomiting. These limitations led to its discontinuation^38^,^39^,^40^. Besides, there is no evidence of its action against any Alphavirus and obviously on CHIKV^38^,^39^,^41^. Substituted benzimidazoles have recently been reported with antiviral action against both RNA and DNA viruses^42^,^43^. Hence, in the present study MBZM-N-IBT was developed and its antiviral property was assessed.

It was observed that MBZM-N-IBT with substituted 2-methyl benzimidazole group, was significantly inhibitory towards CHIKV with minimal cytotoxicity (SI >21) when tested *in vitro* in Vero cells, whereas, MIBT was not at all effective against this virus. To identify the possible antiviral mechanism of MBZM-N-IBT in CHIKV life cycle, the drug was added at different time of post infection. The results suggested that MBZM-N-IBT inhibited CHIKV infection at the early and late phase of replication. The observation of relatively moderate but significant reduction in CHIKV RNA and remarkable reduction in viral protein levels also indicate that MBZM-N-IBT might have multiple targets which ultimately reduce the new viral progeny formation. This is also supported by the efficacy of MBZM-N-IBT at different phases of CHIKV life cycle as observed in time of addition experiment. Additionally, it was observed that MBZM-N-IBT was effective against another strain of CHIKV, DRDE-06. This suggests the potential of MBZM-N-IBT as a drug candidate for further investigation.

Our *in silico* analysis showed affinities of MBZM-N-IBT for CHIKV proteins (3TRK and 3N41) which justified its development as molecular hybrid. In order to assess its potential towards other CHIKV strains, the amino acid residues of 3TRK and 3N41 which showed the possibility of interaction, were looked for their conservation status across several CHIKV strains (isolated from different countries at different times, 38 for nsP2 and 100 for E1). It was observed that these residues were 100% conserved in around 95% strains (Supplementary Table S2.a and S2.b). Hence, it can be speculated that the test compound might have the similar binding affinities for the other CHIKV strains and can be useful as a potential drug for other CHIKV isolates.

In order to explore the binding affinities of MBZM-N-IBT against other RNA viral proteins (Influenza virus H1N1, 3M5R; H5N1, 3F5T strains), this molecular docking study was extended further. The test compound showed good binding affinities with non-structural proteins of H1N1 (3M5R) and H5N1 (3F5T, 2GX9) (Supplementary Table S3). It also showed affinity for C-terminal domain of polymerase basic protein (3KC6) and NS1 effector domain (2GX9) of H5N1. This suggests the possible inhibitory potential of this compound against other RNA viruses which require further experimental validation. Hence, it can be predicted that MBZM-N-IBT might not be very specific for CHIKV and it can still be effective against few other viral diseases.

The actual mechanism of action of MBZM-N-IBT and its effect on host protein is not yet completely clear. Therefore, *in silico* analysis was also conducted to find out possible targets in host cells. The SwissTarget Prediction online tool was used for prediction of off-targets of MBZM-N-IBT in human which may contribute to its possible toxicity^44^,^45^,^46^. The results of this prediction (Supplementary Table S4) revealed that MBZM-N-IBT has low probability (<0.19) as a ligand for the host targets. This is desirable, as this low probability suggest less possibility of host toxicity. However, this prediction is based on structural similarity between known ligands and the query molecule and hence requires further experimental validation. To test the likelihood of MBZM-N-IBT to succeed as a drug, *in silico* prediction of drug likeness was performed by the Molinspiration and SCFBio softwares (www.molinspiration.com, http://www.scfbio-iitd.res.in). The rule of five is considered as drug likeness of any compound and suggests its capacity to be orally active in humans^47^,^48^. The MBZM-N-IBT was found to possess 3 hydrogen bond donor and 6 hydrogen bond acceptor with 4 rotatable bonds. Its log P value was predicted to be 2.445 with a polar surface area of 90.245 Ǻ^2^. Besides, its molar volume and the molar refractivity was found to be 309.756 Ǻ^3^ and 99.957. All these values were within the accepted limits which suggest suitable drug likeness of MBZM-N-IBT. Self-aggregation in the tested media of some thiosemicarbazone derivatives, limit their efficacy^49^. Interestingly, incubation of MBZM-N-IBT in the test media prior to use, did not have any significant influence on its efficacy which is desirable and likely to help in its *in vivo* efficacy.

In this study, it was observed that MBZM-N-IBT inhibits CHIKV infection significantly. Although it abrogates viral RNA and protein synthesis, it is possible that there might be some other complementary mechanisms to reduce viral infection. MBZM-N-IBT may also target some host component. Thus, further studies are required for in-depth exploration of the molecular mechanism of antiviral activity of MBZM-N-IBT in mice model to assess its efficacy.

## Conclusion

In summary, our study showed that MBZM-N-IBT exhibited significant *in vitro* antiviral activity against CHIKV. It inhibited CHIKV even with short exposure and reduced the level of RNA, structural and non-structural proteins. With favourable drug like properties, it has the potential to be a drug candidate against CHIKV, an important pathogen of global health concern. However, further investigation is warranted to evaluate its *in vivo* efficacy and understand its mechanism of action completely.

## Materials and Methods

### Synthesis of compounds

1-[(2-methylbenzimidazol-1-yl) methyl] indoline-2, 3-dione was synthesised by N-mannich reaction between isatin and 2-methyl benzimidazole as described by us earlier^50^ with some modifications. This was further reacted with thiosemicarbazone using schiff base method of preparation with little modification to yield 1-[(2-methylbenzimidazol-1-yl) methyl]-2-oxo-indolin-3-ylidene] amino] thiourea (MBZM-N-IBT) as the test compound^51^. The MIBT is the only IBT class of compounds which was clinically successful against viral diseases; therefore it was used as control. It was synthesized similarly using Schiff base method of preparation with N-methyl isatin and thiosemicarbazone as the starting material. The purification process was performed by repeated washing followed by re-crystallisation. Further purification was accomplished with mini-flash chromatography (Scientific System Inc., Science Park Road State College, PA, USA) using chloroform:methanol:toluene (70:20:10) as solvent system. The purity of the synthesised compounds was checked by HPLC-PDA (Jasco-MD 2010, Ishikawa-machi, Hachioji, Tokyo, Japan) using water:acetonitrile (80:20) solvent system.

The compounds were characterised structurally by high resolution mass spectrometry with a liquid chromatography MS-ion trap-time of flight (micro OTOF-QII, Bruker, Billerica, MA, USA) mass spectrometer and by nuclear magnetic resonance with a Bruker Avance II 400 FT NMR spectrometer (Bruker Biospin, Silberstreifen, Rheinstetten, Germany) (^1^H, 400 MHz, ^13^C, 100 MHz) using deuterated dimethyl sulfoxide as solvent. The FTIR (Jasco- 4100, Ishikawa-machi, Hachioji, Tokyo, Japan) spectra were recorded with potassium bromide pellet.

*MBZM-N-IBT (2).* Yield: 78%. mp: 160–162 °C,^1^H NMR (400 MHz, DMSO): δ (ppm) 12.306 (s, 1H, NH), 8.770 (s, 1H, NH), 7.752–7.170 (m, Ar-H), 5.184 (s, 2H, CH), 2.509 (s, 3H, CH). ^13^C NMR (100 MHz, DMSO): δ (ppm) 178.68 (CS), 161.13 (CO), 142.17 (C, Indole), 131.14 (CN), 130.77 (Ar), 123.32 (Ar), 120.81 (Ar), 119.35 (Ar), 110.64 (C, Indole), 68.92 (CH_2_, Mannich base), 14.74 (CH_3_, Methyl Benzimidazole).FTIR (KBr): ν (cm^−1^) 3427.79 (N-H), 3339.55 (N-H asymm.), 3145.56 (N-H sym.), 2975.15 (Ar-H), 2890 (C-H), 1692.81 (C=O), 1613.76 (C=N).HRMS (m/z): [M + 1]^+^calcd. for C_18_H_16_N_6_OS, 365.1179; found, 365.1094.

*MIBT (1′)*.Yield: 82%. mp: 243–245 °C.^1^H NMR (400 MHz, DMSO): δ (ppm) 12.316 (s, 1H, NH), 8.6655 (s, 1H, NH), 7.6506–7.0576 (m, 4H, Ar-H), 3.3879 (s, 3H, CH). ^13^C NMR (100 MHz, DMSO): δ (ppm) 178.68 (CS), 160.65 (CO), 143.47 (C, Indole), 131.09 (CN), 131.07 (Ar), 122.80 (Ar), 120.50 (Ar), 119.16 (Ar), 109.67 (C, Indole), 25.62 (CH_3_).FTIR (KBr): ν (cm^−1^) 3421.10 (N-H), 3246.57 (N-H asymm.), 3148.22 (N-H sym.), 1679.70 (C=O), 1609.38 (C=N). HRMS (m/z): [M + 1]^+^calcd. for C_10_H_10_N_4_OS, 235.0648; found, 235.0639.

### Cells, virus, antibodies and drugs

Vero cells, originated from African green monkey kidney epithelial cells; S 27, Chikungunya virus prototype strain (accession no. AF369024.2) DRDE06, 2006 Indian ourbreak strain (accession no. EF210157.2) and E2 monoclonal antibody for CHIKV were gifted by Dr. M. M. Parida, DRDE Gwalior, India. Dulbecco’s modified Eagle’s medium (DMEM; PAN Biotech, Aidenbach, Germany) was used to maintain the Vero cell line supplemented with 5% Fetal Bovine Serum (FBS, PAN Biotech, Aidenbach, Germany), Gentamycin and Penicillin-Streptomycin (PAN Biotech, Aidenbach, Germany). The anti-CHIKV-nsP2 monoclonal antibody was developed by us^52^ and GAPDH antibody was procured from Imgenex India (Imgenex, Bhubaneswar, India). MIBT was synthesized by us during this study and Ribavirin was procured from Sigma (Sigma, USA).

### Chikungunya virus infection

Before one day of infection, Vero cells were seeded in 35 mm cell culture dishes (TPP, Trasachingen, Switzerland). Next day, 100% confluent cells were infected with CHIKV S 27 strain with multiplicity of infection (MOI) 0.001 as described earlier^35^. Samples were collected at 15 hours post infection (hpi) according to the assay. The infected cells as well as media, both were harvested for titrating infectious virus particles. Virus infected cells were harvested for checking expression profiles of viral RNA by RT-PCR and viral non-structural proteins by Western blot. Infected cells were examined under microscope (Magnification 20X) and pictures were taken at 15 hpi for the detection of cytopathic effect (CPE).

### Cellular cytotoxicity assay

Approximately, 3000 Vero cells/well were seeded in 96 well plates (Corning) and at 90% confluency, the cells were treated with increasing concentration (from 50 to 800 μM) of MIBT and MBZM-N-IBT along with DMSO as reagent control for 16 hours (h) at 37 °C in CO_2_ Incubator. The cellular cytotoxicity assay was performed as described earlier^36^. In detail, 10 μL MTT reagent (5 mg/mL) (Himedia, Mumbai, India) was added after16 hrs post drug treatment and incubated for 3 hrs at 37 °C. Media was removed, 100 μM DMSO was added and cells were incubated at 37 °C for 15 minutes to dissolve the Formazan crystals. Finally, the absorbance was measured at 550 nm using ELISA plate reader. The metabolically active cell percentage was compared with the control cells and cellular cytotoxicity was determined. This assay was performed three times independently.

### RT-PCR

To quantitate the viral RNA present inside the host cells, Vero cells were infected with S 27 (MOI 0.001). DMSO, Ribavirin (4.1 μM) (Sigma), MIBT (187.8 μM), and different doses of MBZM-N-IBT (50 μM, 100 μM, and 200 μM) were added after infection. The cells were harvested after 15 hpi and the RNA was extracted by using Trizol (Invitrogen, Gaithersburg, MD, USA), the cDNA was generated by using equal amount of RNA (1 μg), random hexamer and first strand cDNA synthesis kit (Fermentas, Vilnius, Lithuania). This cDNA was used to amplify viral non-structural and structural genes (NSP2 and E1)^36^ along with GAPDH as control.

### Flow Cytometric Analysis (FACS)

The CHIKV infected and mock Vero cells with or without different drugs were detached from cell culture dishes by trypsin EDTA treatment. Cells were then fixed in 4% paraformaldehyde for ten minutes at room temperature. For intra cellular staining (ICS) of CHIKV antigens, cells were permeabilized in permeabilization buffer (1X PBS + 0.5% BSA + 0.2% Saponin + 0.01% NaN_3_) followed by blocking in 1% BSA (in permeabilization buffer) for 30 min at RT. Then cells were incubated in anti nsP2 and E2 in 1X permeabilization buffer for 30 minutes at RT. Cells were washed twice in 1X permeabilization buffer to remove unbound antibodies followed by incubation in Alexa Fluor (AF) 488 conjugated chicken anti-mouse antibody (Invitrogen, USA). Approximately 1×10^4^ cells were acquired by FACSCalibur^TM^ flow cytometer (BD Biosciences, San Jose, California, USA) for each sample and analyzed by CellQuest Pro software (BD Biosciences, San Jose, California, USA). Statistical analysis was performed by using GraphPad Prism version 5. p < 0.05 was considered as statistically significant.

### Western blot

As described earlier^52^, Virus infected and drug treated Vero cells were harvested at 15 hpi according to the procedure mentioned above. Cells were lysed using equal volume of RIPA buffer and protein expression profiles were assessed by the protocol described earlier^35^. After quantitation, 60 μg of protein was run in 10% SDS-polyacrylamide gel and separated proteins were transferred on to PVDF membrane. To check the viral non-structural proteins, PVDF membrane was probed with nsP2 (1:3000 dilution) and E2 (1:3000 dilution) monoclonal antibodies and the same membrane was probed for GAPDH to use as loading control. Blots were developed by developing reagents NBT/BCIP (Promega, Madison, WI, USA). The intensities of all the viral non-structural protein bands were quantified from three independent experiments using Quantity One software (Bio Rad, GmbH, Munchen).

### Plaque assay

To quantitate the infectious viral titre, plaque assay was performed according to previously mentioned procedure^52^. The infected and drug treated samples (Vero cells and the supernatants) were harvested at 15 hpi. Each sample was serially diluted (from 10^−1^ to 10^−5^) in serum free media and used to infect the 100% confluent Vero cells in 6 well cell culture dishes. After 1 and half hour shaking, the cells were washed to remove the unattached viruses and the cells were overlaid with methyl cellulose (Sigma, St. Louis, MO, USA) containing DMEM and kept at 37 °C in CO_2_ incubator for 3 to 4 days for visible plaques to develop. The cells were fixed with 8% formaldehyde for 4 hours and stained. The number of plaques was counted as Plaque Forming Unit/ml (PFU/ml) and the bar diagram was generated using Prism software. The plaques were counted from two dishes for each experiment and the data represent the mean of three independent experiments (in duplicate) ±standard deviation (SD).

### Molecular docking

The molecular docking of MBZM-N-IBT and MIBT was carried out following earlier reported method^37^,^53^. In brief, X-ray crystallographic structure of CHIKV nsP2 protease (3TRK) and mature envelope glycoprotein complex (spontaneous cleavage, 3N41) were recovered from protein data bank. The non-structural protein models developed by homology modelling^54^ of CHIKV nsP1, nsP3, and nsP4 were also taken for the study. The structures of proteins (3TRK, and 3N41) were optimised by extraction of any co-crystallized ligand and water molecules from the catalytic site using the Discovery studio visualise package (Discovery studio 3.5). The ligand geometry was optimised using the Argus Lab package (Argus Lab 4.0.1) and docked to the macromolecule using the AutoDock vina to calculate binding affinity and the binding affinity <−6 Kcal/mol was considered as significant for the drug-target complex^37^,^55^. Structural components of MBZM-N-IBT were taken as decoys to validate the study. The interaction between ligand and macromolecule was visualised using the PyMOL molecular graphics system (PyMOL 1.3).

### RNA extraction and RT-qPCR

Viral RNA was extracted from 140 ul of infected and drug treated cells and culture supernatants by using the QIAamp Viral RNA Mini Kit (Qiagen, Hilden, Germany) as per manufacturer’s protocol. Further, RNA was converted to cDNA by first strand cDNA synthesis kit with random hexamer primer (Fermentas, USA). The cDNA was used for real-time PCR assay with CHIKV specific PCR primers (CL011(F)-5′TGCCGTCACAGTTAAGGACG3′ and CL012(R)-5′CCTCGCATGACATGTCCG3′^56^,^57^ directed to the envelope-E1 gene) using MESA GREEN qPCR master mix plus for SYBR assay (Eurogentec, Belgium). The quantitative RT-PCR was carried out in Opticon 3 system (MJ Research, Canada). Samples were assayed in a 20 μL reaction volume containing 10 μL of 2× Master mix, 0.5 μL (5pmol) each of forward and reverse primers, 7.5 μL of nuclease free water and 1 μL of RNA. The thermal profile consisted of 40 cycles of PCR at 95 °C for 45 s, 62 °C for 30 s, and 72 °C for 30 s. Positive and negative template control was also included in all experiments. Each sample was analyzed in triplicates.

### Statistical Analysis

The statistical analyses were performed by using One-way ANOVA (nonparametric, and Kruskal-Wallis test) in Graph Pad Prism 5.0 Software. The statistical analysis of the experimental data was presented as mean ± SD of three independent experiments (n ≥ 3).P-value less than 0.001 were considered to be statistically significant in the tests.

## Additional Information

**How to cite this article**: Mishra, P. *et al.* Inhibition of Chikungunya Virus Replication by 1-[(2-Methylbenzimidazol-1-yl) Methyl]-2-Oxo-Indolin-3-ylidene] Amino] Thiourea (MBZM-N-IBT). *Sci. Rep.* **6**, 20122; doi: 10.1038/srep20122 (2016).

## Supplementary Material

Supplementary Information

## Figures and Tables

**Table 1:** *In-silico* binding affinity of compounds for structural and non-structural proteins of CHIKV.

Ligand	Macromolecules	
	3N41	3TRK
MBZM-N-IBT	−7.4	−7.4
MIBT	−5.9	−6.4
IBT	−6.7	−6.7
2-methyl benzimidazole	−5.4	−5.4
Isatin	−5.8	−5.7
Thiosemicarbazone	−3.5	−3.6

Binding affinity in Kcal/mol with root mean square deviation of upper bound and lower bound as zero. 3N41: mature envelope glycoprotein complex (spontaneous cleavage) of CHIKV. 3TRK: nsP2 protease of CHIKV.
