# Supplementary material for: Inhibition of Chikungunya Virus Replication by 1-[(2-Methylbenzimidazol-1-yl) Methyl]-2-Oxo-Indolin-3-ylidene] Amino] Thiourea(MBZM-N-IBT)
Source: Sci Rep. 2016 Feb 4;6:20122. doi: 10.1038/srep20122 (PMC4740769; doi:10.1038/srep20122)

Inhibition of Chikungunya Virus Replication by 1-[(2-Methylbenzimidazol-1-yl) Methyl]-2-Oxo-Indolin-3-ylidene] Amino] Thiourea (MBZM-N-IBT)

Priyadarsee Mishra[#1], Abhishek Kumar [#2], Prabhudutta Mamidi[2], Sameer Kumar[2], Itishree Basantray[2], Tanuja Saswat[2], Indrani Das[2], Tapas Kumar Nayak[3], Subhasis Chattopadhyay[3], Bharat Bhusan Subudhi[1*], Soma Chattopadhyay[2*]

[#] Equal contribution

[1]School of Pharmaceutical Sciences, Siksha O Anusandhan University, Bhubaneswar, India

[2]Institute of Life Sciences, Bhubaneswar, India

[3]School of Biological Sciences, National Institute of Science Education & Research, Bhubaneswar, India

[*]**Address of Corresponding author:**

Soma Chattopadhyay

Institute of Life Sciences,

Nalco Square, Bhubaneswar-751023, India

Phone No: 0091 674 2301676; Fax No: 0091 674 2300728

Email: sochat.ils@gmail.com

and

Bharat Bhusan Subudhi

School of Pharmaceutical Sciences, Siksha O Anusandhan University

Khandagiri Square, Bhubaneswar-751003, India

Phone No: 09853945363; Email: bharatbhusans@gmail.com

Supplementary figure S1: Characterization of MBZM-N-IBT. (**a**). Chromatogram was obtained with chloroform: methanol: toluene (70:20:10) as the mobile phase in HPLC with PDA detector. (**b**). FTIR spectrum of MBZM-N-IBT. The spectrum was recorded with KBr pellet in FTIR. (**c**).^{1}H NMR spectrum of MBZM-N-IBT. The spectrum was recorded with DMSO-d6 as solvent at 400 MHz. (**d**).^{13}C NMR spectrum of MBZM-N-IBT. The spectrum was recorded with DMSO-d6 as solvent at 100 MHz.

Supplementary Figure S2: **Characterization of MIBT**. **(a)**. Chromatogram was obtained with chloroform: methanol: toluene (70:20:10) as the mobile phase in HPLC with PDA detector. **(b).** FTIR spectrum of MIBT. The spectrum was recorded with KBr pellet in FTIR. **(c).** ^{1}H NMR spectrum of MIBT. The spectrum was recorded with DMSO-d6 as solvent at 400 MHz. **(d)**. ^{13}C NMR spectrum of MIBT. The spectrum was recorded with DMSO-d6 as solvent at 100 MHz.

Supplementary Figure S3: Quantitation of Viral RNA from cell culture supernatant. Vero cells were infected with S 27 and drug treated as mentioned in figure 4(b). The supernatants were collected at 15 hpi, viral RNA was extracted and RT-qPCR was performed. Graph showing the C_T value as mean ± SEM from three independent experiments.

Supplementary Figure S4: Inhibitor dose response curve. Vero cells were infected with either S27 or DRDE-06 virus strains and MBZM-N-IBT was added with different concentrations. Supernatant was collected after 15 hpi and virus titer was determined by plaque assay. Graph showing the virus titer as mean ± SEM from three independent experiments. (a) Represent the EC_{50} value of S 27 and (b) represent DRDE-06.

Supplementary Figure S5: Inhibition of DRDE-06 replication in presence of MBZM-N-IBT. Vero cells were infected with S 27 or DRDE-06 and treated with different concentrations of MBZM-N-IBT. Supernatants were collected and viral titers were

determined by plaque assay. Data represents the log 10 of virus titer as mean ± SEM from three independent experiments.

Supplementary Figure S6: Flow cytometric analysis depicting inhibition of CHIKV nsP2 and E2 protein expression after treatment with MBZM-N-IBT: (a). Mean Fluorescence Intensity (MFI) plot of nsP2 and E2 representing isotype (purple filled), mock + DMSO (dark dashed line), S 27+DMSO (green solid line), S 27+ MBZM-N-IBT (50; red solid line, 100; dark solid line and 200 µM; blue solid line). **(b, c).** Graphical representation depicting nsP2 and E2 MFI. Data represented as mean ± SEM from three independent experiments.

Supplementary Table S1. In-silico binding affinities of MBZM-N-IBT and its decoys with homologous models of CHIKV nsP1, nsP2, nsP3 and nsP4 proteins. Binding affinity in Kcal/mol with root mean square deviation of upper bound and lower bound as zero.

**Ligand**	**Macromolecules**			
	**nsP1**	**nsP2**	**nsP3**	**nsP4**
MBZM-N-IBT	-7.3	-8.9	-7.9	-8.3
MIBT	-6.8	-7.4	-6.4	-6.9
IBT	-6.9	-7.1	-7.1	-7.4
2-methyl benzimidazole	-5.4	-6.2	-6.2	-6.4
Isatin	-6.2	-6.9	-6.9	-7.0
Thiosemicarbazone	-3.7	-3.8	-3.7	-4.0

Table S2a. Conservation status of amino acids (residues) of MBZM-N-IBT-E1/E2 (3N41) complex in different strains of CHIKV

Accession of CHIKV strain downloaded from NCBI database	152	183	185	247	248	250
**AAN05102.2 SP CHIKV**	**P**	**D**	**Y**	**R**	**G**	**S**
ACA81773.1 SP CHIKV	.	.	.	.	.	.
ACB58723.1 SP CHIKV	.	.	.	.	.	.
ACM09909.1 SP CHIKV	.	.	.	.	.	.
ACM09911.1 SP CHIKV	.	.	.	.	.	.
ACM09915.1 SP CHIKV	.	.	.	.	.	.
ACM09917.1 SP CHIKV	.	.	.	.	.	.
ACM09919.1 SP CHIKV	.	.	.	.	.	.
ACM09921.1 SP CHIKV	.	.	.	.	.	.
ACS45303.1 SP CHIKV	.	.	.	.	.	.
ACT10814.1 E1 CHIKV	.	V	.	.	.	.
ACV88657.1 SP CHIKV	.	.	.	.	.	.
ACY09939.1 SP CHIKV	.	.	.	.	.	.
ACY25938.1 SP CHIKV	.	.	H	.	.	.
ACY25942.1 SP CHIKV	.	.	.	.	.	.
ACY25946.1 SP CHIKV	.	.	.	.	.	E
ACY66830.1 SP CHIKV	.	.	.	.	.	.
ACY66836.1 SP CHIKV	.	.	.	.	.	.
ACY66838.1 SP CHIKV	.	.	.	.	.	.
ACY66839.1 SP CHIKV	.	.	.	.	.	.
ACY66841.1 SP CHIKV	.	.	.	.	.	.
ACY66843.1 SP CHIKV	.	.	.	.	.	.
ACY66845.1 SP CHIKV	.	.	.	.	.	.
ACY66847.1 SP CHIKV	.	.	.	.	.	.
ACZ72971.1 SP CHIKV	.	.	.	.	.	.
ACZ98835.1 SP CHIKV	.	.	.	.	.	.
ADC53730.1 SP CHIKV	.	.	.	.	.	.
ADC53731.1 SP CHIKV	.	.	.	.	.	.
ADC53733.1 SP CHIKV	.	.	.	.	.	.
ADC84390.1 E1 CHIKV	.	.	.	.	.	.
ADG95881.1 SP CHIKV	.	.	.	.	.	.
ADG95883.1 SP CHIKV	.	.	.	.	.	.
ADG95885.1 SP CHIKV	.	.	.	.	.	.
ADG95887.1 SP CHIKV	.	.	.	.	.	.
ADG95889.1 SP CHIKV	.	.	.	.	.	.
ADG95891.1 SP CHIKV	.	.	.	.	.	.
ADG95893.1 SP CHIKV	.	.	.	.	.	.
ADG95895.1 SP CHIKV	.	.	.	.	.	.
ADG95897.1 SP CHIKV	.	.	.	.	.	.
ADG95899.1 SP CHIKV	.	.	.	.	.	.
ADG95901.1 SP CHIKV	.	.	.	.	.	.
ADG95902.1 SP CHIKV	.	.	.	.	.	.
ADG95904.1 SP CHIKV	.	.	.	.	.	.
ADG95906.1 SP CHIKV	.	.	.	.	.	.
ADG95907.1 SP CHIKV	.	.	.	.	.	.
ADG95909.1 SP CHIKV	.	.	.	.	.	.
ADG95911.1 SP CHIKV	.	.	.	.	.	.
ADG95913.1 SP CHIKV	.	.	.	.	.	.
ADG95915.1 SP CHIKV	.	.	.	.	.	.
ADG95917.1 SP CHIKV	.	.	.	.	.	.
ADG95919.1 SP CHIKV	.	.	.	.	.	.
ADG95921.1 SP CHIKV	.	.	.	.	.	.
ADG95923.1 SP CHIKV	.	.	.	.	.	.
ADG95924.1 SP CHIKV	.	.	.	.	.	.
ADG95926.1 SP CHIKV	.	.	.	.	.	.
ADG95928.1 SP CHIKV	.	.	.	.	.	.
ADG95930.1 SP CHIKV	.	.	.	.	.	.
ADG95932.1 SP CHIKV	.	.	.	.	.	.
ADG95934.1 SP CHIKV	.	.	.	.	.	.
ADG95936.1 SP CHIKV	.	.	.	.	.	.
ADG95938.1 SP CHIKV	.	.	.	.	.	.
ADG95940.1 SP CHIKV	.	.	.	.	.	.
ADG95942.1 SP CHIKV	.	.	.	.	.	.
ADG95944.1 SP CHIKV	.	.	.	.	.	.
ADG95946.1 SP CHIKV	.	.	.	.	.	.
ADG95948.1 SP CHIKV	.	.	.	.	.	.
ADG95950.1 SP CHIKV	.	.	.	.	.	.
ADG95952.1 SP CHIKV	.	.	.	.	.	.
ADG95954.1 SP CHIKV	.	.	.	.	.	.
ADG95956.1 SP CHIKV	.	.	.	.	.	.
ADI88515.1 SP CHIKV	.	.	.	.	.	.
ADJ19188.1 SP CHIKV	.	.	.	.	.	.
ADJ19190.1 SP CHIKV	.	.	.	.	.	.
ADK24722.1 SP CHIKV	.	.	.	.	.	.
ADN85586.1 E1 CHIKV	.	.	.	.	.	.
ADN85587.1 E1 CHIKV	.	.	.	.	.	.
ADN85594.1 E1 CHIKV	.	.	.	.	.	.
ADQ37313.1 SP CHIKV	.	.	.	.	.	.
ADQ37314.1 SP CHIKV	.	.	.	.	.	.
ADZ04936.1 SP CHIKV	.	.	.	.	.	.
AEA10291.1 SP CHIKV	.	.	.	.	.	.
AEE60791.1 SP CHIKV	.	.	.	.	.	.
AEE60792.1 SP CHIKV	.	.	.	.	.	.
AEE60794.1 SP CHIKV	.	.	.	.	.	.

AEE60797.1 SP CHIKV	.	.	.	.	.	.
AEJ18139.1 E1 CHIKV	.	.	.	.	.	.
AEK21840.1 SP CHIKV	.	.	.	.	.	.
AEK31253.1 SP CHIKV	.	.	.	.	.	.
AEX25334.1 SP CHIKV	.	.	.	.	.	.
AFD61558.1 SP CHIKV	.	.	.	.	.	.
AFM35612.1 SP CHIKV	.	.	.	.	.	.
AFM38219.1 SP CHIKV	.	.	.	.	.	.
AFP43244.1 SP CHIKV	.	.	.	.	.	.
BAH97931.1 SP CHIKV	.	.	.	.	.	.
BAH97933.1 SP CHIKV	.	.	.	.	.	.
CAX63320.1 SP CHIKV	.	.	.	.	.	.
CCA61128.1 E2-6K-E1 CHIKV	.	.	.	.	.	.
CCA61131.1 E2-6K-E1 CHIKV	.	.	.	.	.	.

Table S2b. Conservation status of amino acids (residues) of MBZM-N-IBT-nsP2 protease domain (3TRK) complex in different strains of CHIKV

S.N.	Accession No.	Strain Name	Year	Place of Isolation	1013	1014	1047	1050	1079	1083
**1**	**AAN05101.1**	**S 27 (African prototype)**	**1954**	**Tanzania**	**C**	**W**	**Y**	**E**	**Y**	**H**
2	AAU43880.1	Senegal_37997_ 1983	1983	Senegal	.	.	.	.	.	.
3	CAJ90474.1	La Reunion_06-021_2005	2005	Reunion Island	.	.	.	.	.	.
4	ABD95937.2	LR2006 OPY1 (Re-Union)	2006	Reunion Island	.	.	.	.	.	.
5	ABJ98543.1	D570/06 (Mauritius)	2006	Mauritius	.	.	.	.	.	.
6	ABN04187.1	IND-06-AP3	2006	India	.	.	.	.	.	.
7	ABN04189.1	IND-06-KA15	2006	India	.	.	.	.	.	.
8	ABN04191.1	IND-06-MH2	2006	India	.	.	.	.	.	.
9	ABN04193.1	IND-06-RJ1	2006	India	.	.	.	.	.	.
10	ABN04195.1	India_TN1_2006	2006	India	.	.	.	.	.	.
11	ABN04197.1	IND-00-MH4 (Yawat)	2000	India	.	.	.	.	.	.
12	ABN04201.1	India_MH5_1973	1973	India	.	.	.	.	.	.
13	ABP88821.1	DRDE-06	2006	India	.	.	.	.	.	.
14	ABU93704.1	Wuerzburg (Mauritius)	2006	Mauritius	.	.	.	.	.	.
15	ABX38963.1	ITA07-RA1(Long Varient)	2007	Italy	.	.	.	.	.	.
16	ACD93567.1	MY002IMR/06/BP	2006	Malaysia	.	.	.	.	.	.
17	ACD93607.1	TM25 (Mauritius)	2006	Mauritius	.	.	.	.	.	.
18	ACD93609.1	CHIK31 (Rajasthan)	2006	India	.	.	.	.	.	.
19	BAH97930.1	Japan_SL11131_2006	2006	Japan	.	.	.	.	.	.
20	ACY25937.1	KL06,RGCB03	2006	India	.	.	.	.	.	.
21	ACY25939.1	KL06	2006	India	.	.	.	.	.	.
22	ACY25941.1	KL07	2007	India	.	.	.	.	.	.
23	ACY25943.1	KL07	2007	India	.	.	.	.	.	.
24	ACY25945.1	KL08	2008	India	.	.	.	.	.	.
25	ACY66840.1	Singapore_0611aTw_2006	2006	Singapore	.	.	.	.	.	.
26	ACY66844.1	Bangladesh_0810aTw_2008	2008	Bangladesh	.	.	.	.	.	.
27	ACY66846.1	Malaysia_0810bTw_2008	2008	Malaysia	.	.	.	.	.	.
28	ADG95884.1	Nigerai_IbH35_1964	1964	Nigeria	.	.	.	.	.	.
29	ADG95905.1	Indonesia_RSU1_1985	1985	Indonesia	.	.	.	.	.	.
30	ADG95912.1	Sri-Lanka_CK1_2007	2007	Sri-Lanka	.	.	.	.	.	.
31	ADG95927.1	Congo_LSFS_1960	1960	Congo	.	.	.	.	.	.
32	ADG95929.1	Thailand_TH35_1958	1958	Thailand	.	.	.	.	.	.
33	ADG95931.1	Tanzania_Ross Low-psg_1953	1953	Tanzania	.	.	.	.	.	.
34	ADG95933.1	Uganda_Ag4155_1982	1982	Uganda	.	.	.	.	.	.
35	ADG95935.1	India_Gibbs263_1963	1963	India	.	.	.	.	.	.
36	ADG95937.1	Thailand_1455_1975	1975	Thailand	.	.	.	.	.	.
37	ADJ19189.1	Thailand_CU10_2008	2008	Thailand	.	.	.	.	.	.
38	ADJ19191.1	Thailand_CU683_2009	2009	Thailand	.	.	.	.	.	.
39	3TRK From PDB				.	.	.	.	.	.

Supplementary Table S3. Binding affinity of MBZM-N-IBT against potential targets in Alphavirus and RNA virus

PDB ID	Target	Binding affinity(Kcal/mol)
3M5R	NS1 effecter domain(H1N1, Swine flu)	-7.3
3F5T	NS1(H5N1)	-6.5
3KC6	C-terminal domain of polymerase basic protein(H5N1)	-7.7
2GX9	NS1 effecter domain(H5N1)	-9.0
3MUU	E2-E1 heterodimer (Sindbis virus)	-8.5
1RER	Fusion glycoprotein E1 (Semliki forest virus)	-7.6

Supplementary Table S4. Probability of off-targets of MBZM-N-IBT in Homo sapiens

Target	Uniprot ID	Gene Code	ChEMBL ID	By Homology	Probability	Target Class
Microtubule-associated protein tau	P10636	MAPT	CHEMBL1293224	No	0.19	Unclassified
Cannabinoid receptor 1	P21554	CNR1	CHEMBL218	No	0.18	Membrane receptor
Cannabinoid receptor 2	P34972	CNR2	CHEMBL253	No	0.18	Membrane receptor
Hepatocyte growth factor receptor	P08581	MET	CHEMBL3717	No	0.18	Tyr Kinase
Cyclin-dependent kinase 1	P06493	CDK1	CHEMBL308	No	0.16	Ser_Thr Kinase
Cyclin-dependent kinase 4	P11802	CDK4	CHEMBL331	Yes	0.16	Ser_Thr Kinase
Cyclin-dependent kinase 2	P24941	CDK2	CHEMBL301	No	0.16	Ser_Thr Kinase
Cyclin-dependent kinase 6	Q00534	CDK6	CHEMBL2508	Yes	0.16	Ser_Thr Kinase
Cyclin-dependent kinase 3	Q00526	CDK3	CHEMBL4442	Yes	0.16	Ser_Thr Kinase
FAD-linked sulfhydryl oxidase ALR	P55789	GFER	CHEMBL1741189	No	0.14	Enzyme
Beta-secretase 1	P56817	BACE1	CHEMBL4822	No	0.13	Aspartic Protease
Beta-secretase 2	Q9Y5Z0	BACE2	CHEMBL2525	Yes	0.13	Aspartic Protease
Tyrosine-protein phosphatase non-receptor type 1	P18031	PTPN1	CHEMBL335	No	0.13	Tyr Phosphatase
Tyrosine-protein phosphatase non-receptor type 2	P17706	PTPN2	CHEMBL3807	Yes	0.13	Tyr Phosphatase
Melanin-concentrating hormone receptor 1	Q99705	MCHR1	CHEMBL344	No	0.11	Membrane receptor

Supplementary Figure S1a

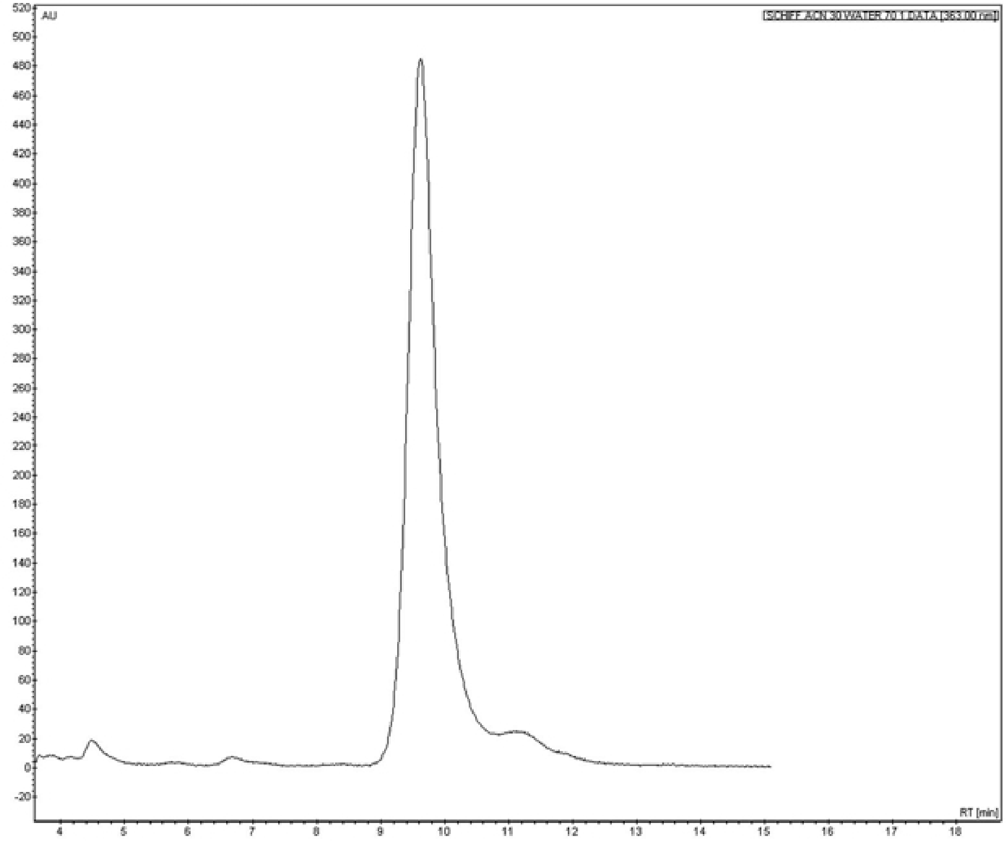

Supplementary Figure S1b

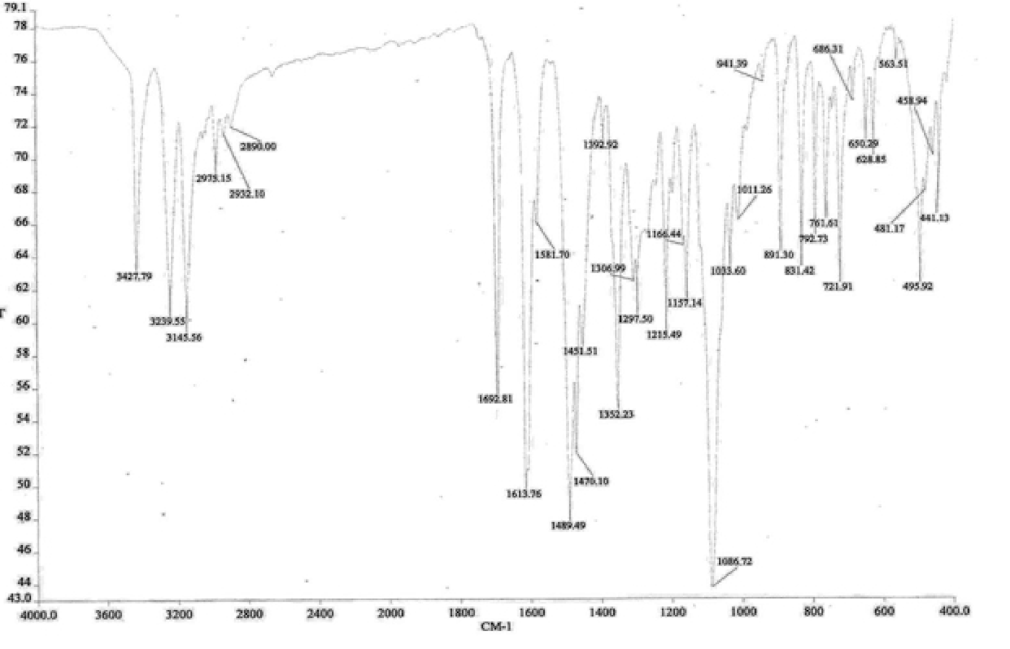

Supplementary Figure S1c

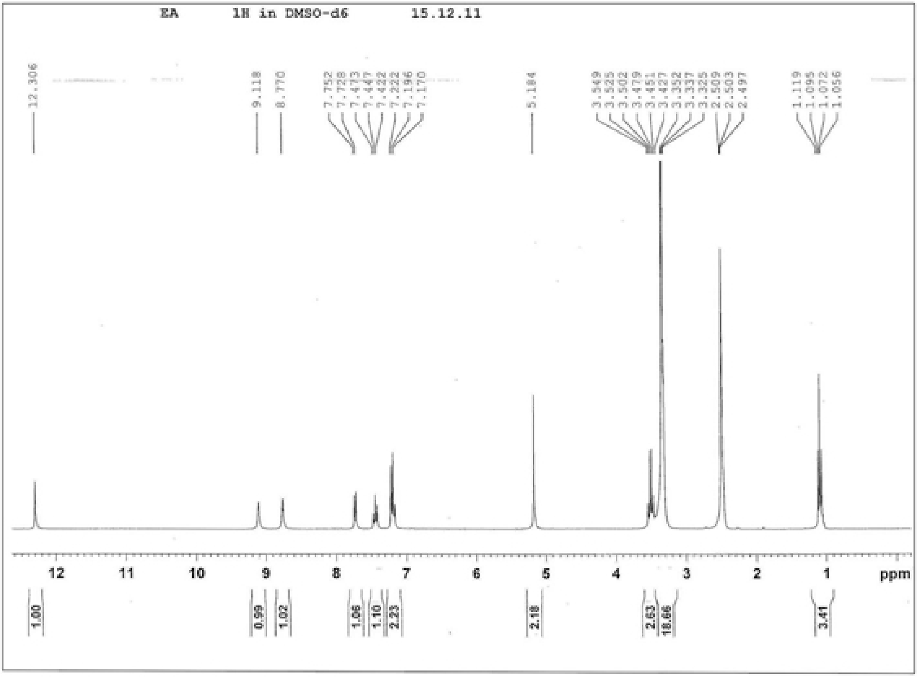

Supplementary Figure S1d

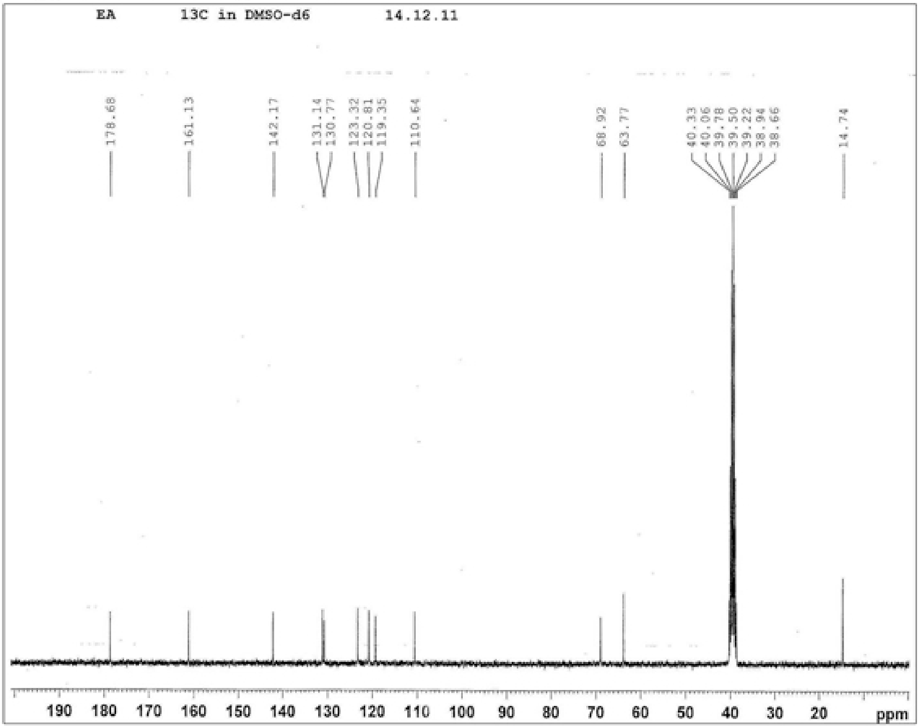

Supplementary Figure S2a

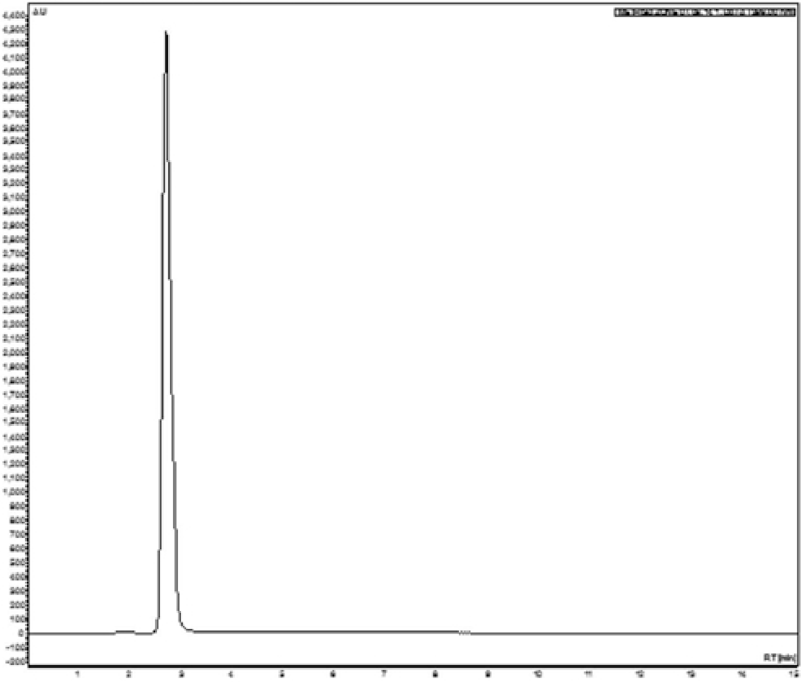

Supplementary Figure S2b

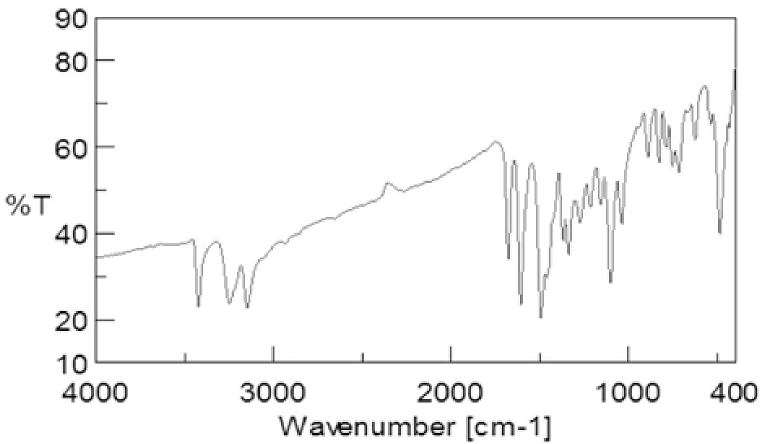

Supplementary Figure S2c

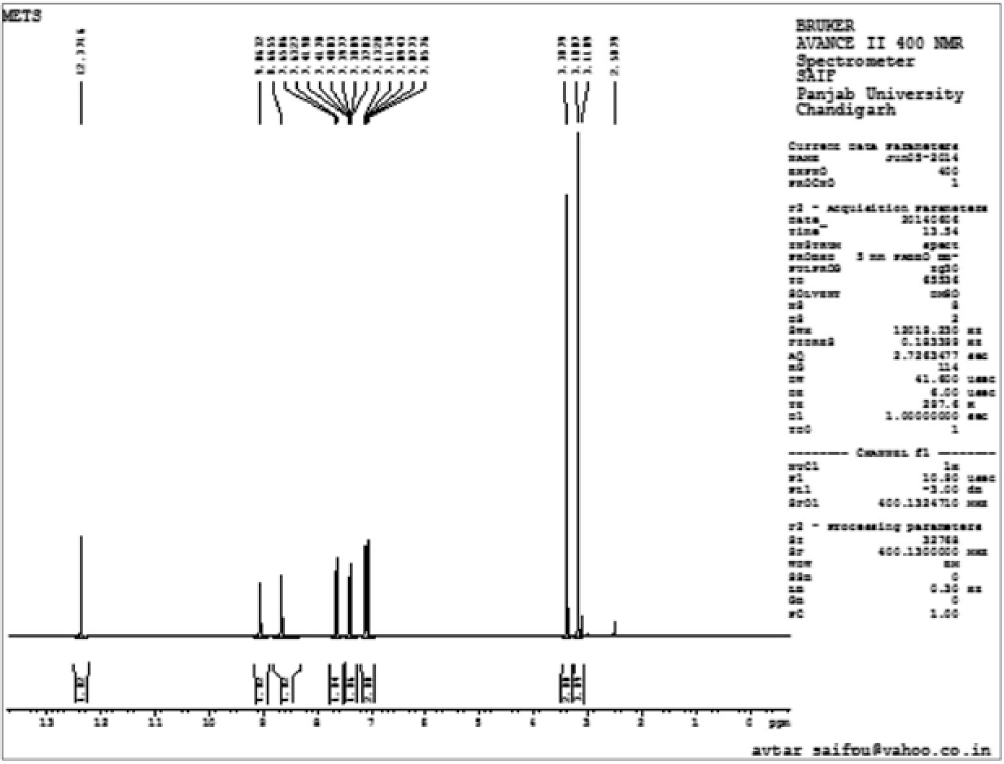

Supplementary Figure S2d

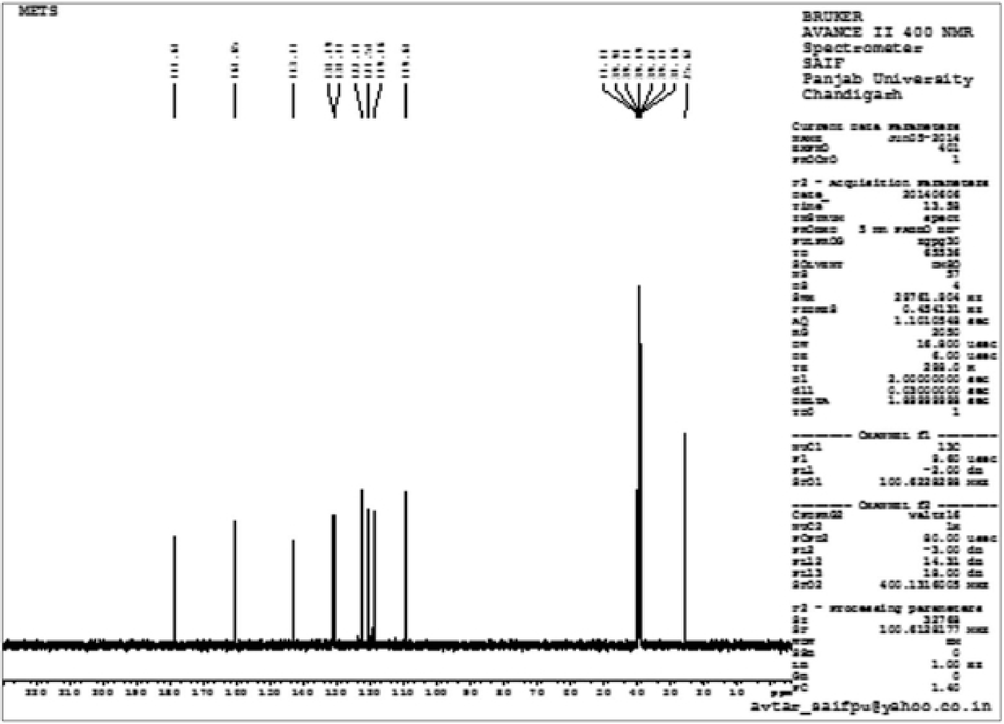

Supplementary Fig. 3

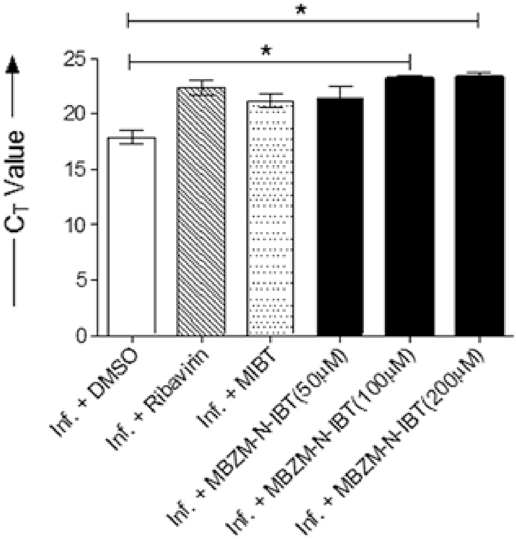

Supplementry Fig. 4 (a) (b)

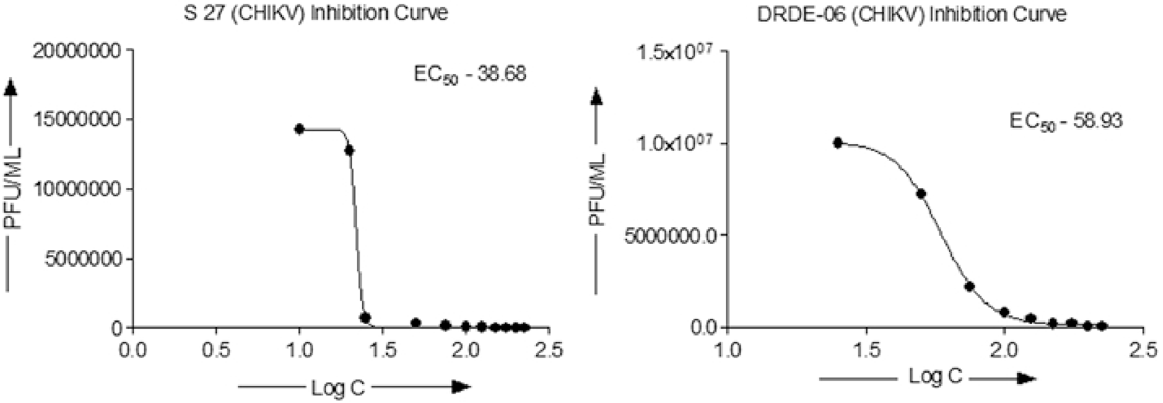

Supplementary Fig.5

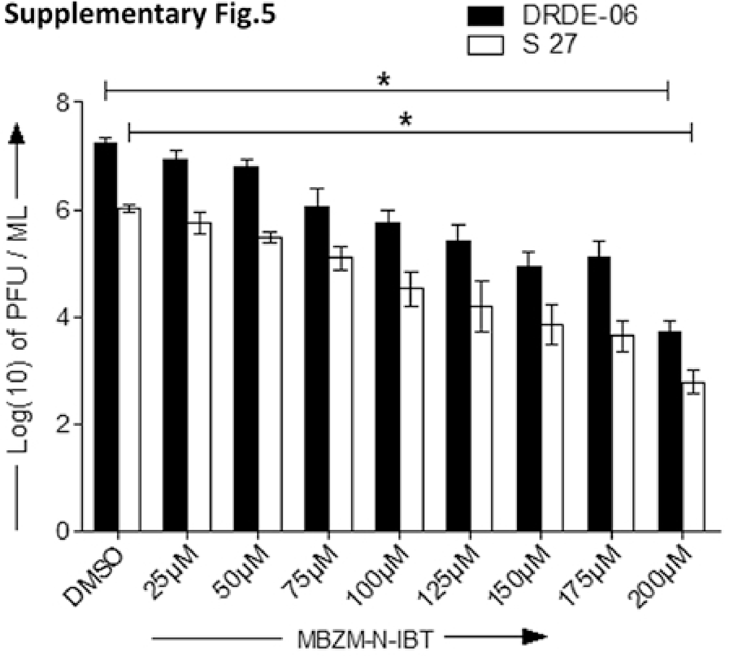

Supplementary Figure 6. (a)

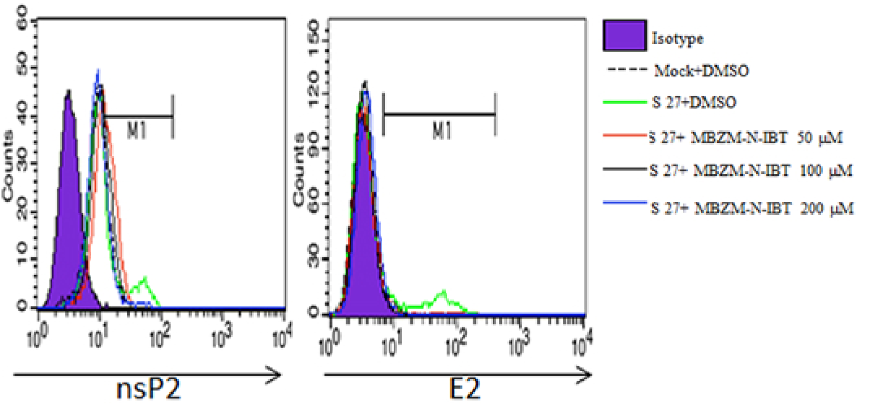

(b)

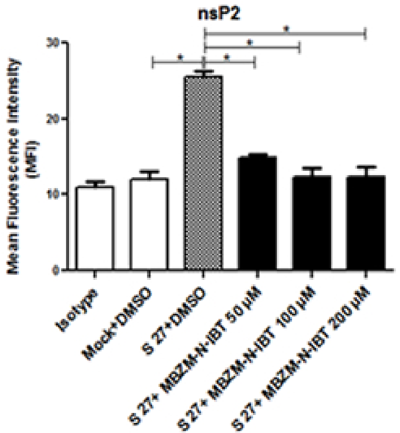

(c)

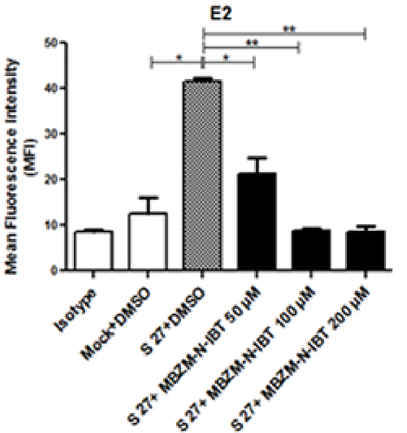

Supplement: Supplementary Information [file srep20122-s1.pdf]
